# Structure and function analysis of the essential 3′X domain of hepatitis C virus

**DOI:** 10.1261/rna.073189.119

**Published:** 2020-02

**Authors:** Jesús Castillo-Martínez, Tamara Ovejero, Cristina Romero-López, Isaías Sanmartín, Beatriz Berzal-Herranz, Elisa Oltra, Alfredo Berzal-Herranz, José Gallego

**Affiliations:** 1 Facultad de Medicina, Universidad Católica de Valencia, Valencia, 46001, Spain; 2 Escuela de Doctorado, Universidad Católica de Valencia, Valencia, 46001, Spain; 3 Instituto de Parasitología y Biomedicina “López-Neyra” (IPBLN-CSIC), Armilla, Granada, 18016, Spain

**Keywords:** 3'X domain, 5BSL3.2 domain, hepatitis C virus, RNA structure, replication cycle

## Abstract

The 3′X domain of hepatitis C virus has been reported to control viral replication and translation by modulating the exposure of a nucleotide segment involved in a distal base-pairing interaction with an upstream 5BSL3.2 domain. To study the mechanism of this molecular switch, we have analyzed the structure of 3′X mutants that favor one of the two previously proposed conformations comprising either two or three stem–loops. Only the two-stem conformation was found to be stable and to allow the establishment of the distal contact with 5BSL3.2, and also the formation of 3′X domain homodimers by means of a universally conserved palindromic sequence. Nucleotide changes disturbing the two-stem conformation resulted in poorer replication and translation levels, explaining the high degree of conservation detected for this sequence. The switch function attributed to the 3′X domain does not occur as a result of a transition between two- and three-stem conformations, but likely through the sequestration of the 5BSL3.2-binding sequence by formation of 3′X homodimers.

## INTRODUCTION

**FIGURE 1.**
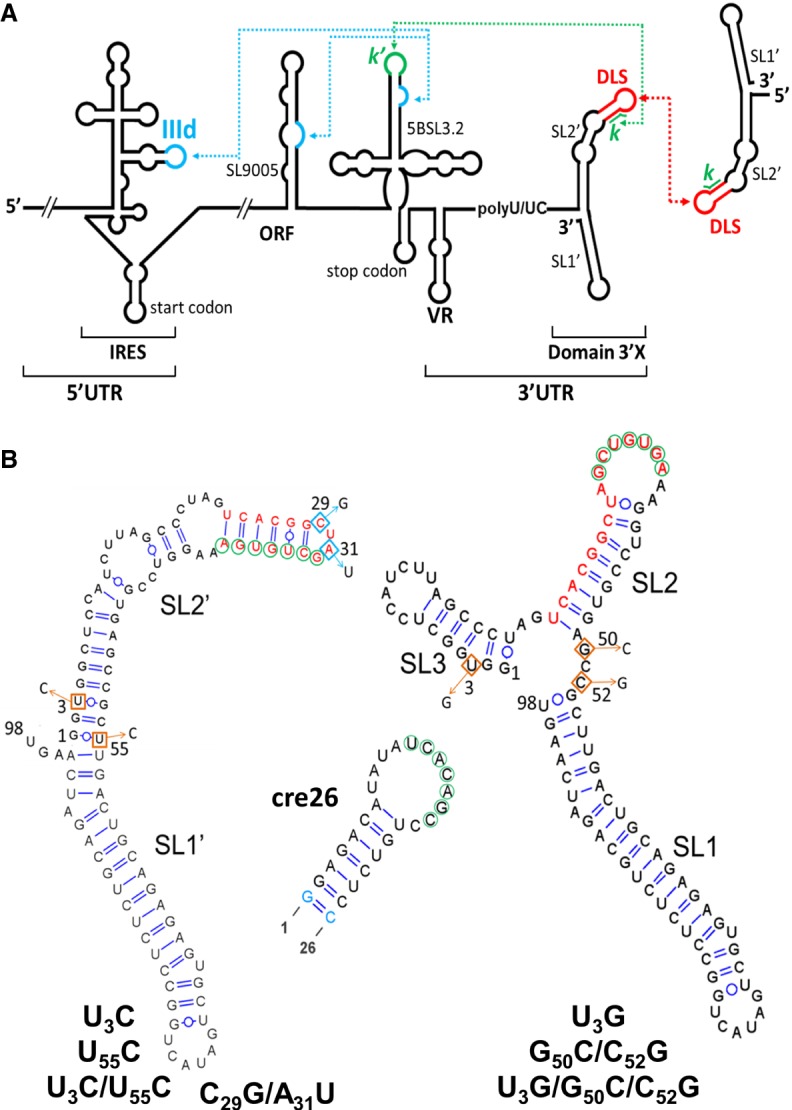
Secondary structure of HCV 3′X wild-type and mutant domains analyzed in this study. (*A*) Schematic representation of the secondary structure of the 5′ and 3′ regions of the HCV RNA genome. Functional RNA–RNA interactions are indicated by blue and green dotted arrows. The red arrow represents an intermolecular contact between palindromic DLS sequences that promotes viral RNA homodimerization. (*B*) Predicted secondary structure of the 98-nt-long 3′X domain and the 26-nt-long apical portion of the 5BSL3.2 domain (cre26). The U_3_C, U_55_C, and U_3_C/U_55_C 3′X domain mutants stabilize the two-stem conformation observed for the wild-type sequence, whereas the U_3_G, G_50_C/C_52_G, and U_3_G/G_50_C/C_52_G mutants were designed to favor an alternative domain structure formed by three stems. The C_29_G/A_31_U mutant, previously shown to adopt the two-stem conformation (Cantero-Camacho and Gallego 2018), contains a double mutation that disrupts the DLS palindrome and blocks 3′X domain dimerization. The blue-colored nt in cre26 indicates changes relative to the wild-type sequence introduced to increase transcription yield. The *k* sequence motif within domain 3′X (indicated with green circles) has been shown to establish a distal interaction involving Watson–Crick pairs with a complementary *k*′ sequence (green circles) in the apical loop of 5BSL3.2. The 16-nt palindromic DLS motif (depicted with red nt) includes the *k* tract and is universally conserved among all HCV isolates. The two- and three-stem conformations of the 3′X domain expose differently the *k* and DLS motifs.

The Hepatitis C virus (HCV) is an important human pathogen that currently affects around 71 million people worldwide, according to estimates from the World Health Organization. The genomic RNA of HCV comprises a single ORF flanked at either end by untranslated regions (UTRs) containing structured domains that play key roles in the viral cycle. The 5′-UTR is mostly occupied by an internal ribosome entry site (IRES) involved in the initiation of viral protein synthesis (Johnson et al. 2017), whereas the 3′-UTR region contains a highly conserved 98-nt-long domain named 3′X (Tanaka et al. 1995; Kolykhalov et al. 1996) that is essential for replication (Kolykhalov et al. 2000; Friebe and Bartenschlager 2002; Yi and Lemon 2003a,b; Masante et al. 2015). The IRES and the 3′X region are connected through domain 5BSL3.2, which establishes a network of distal RNA–RNA contacts involving both the IRES and domain 3′X. 5BSL3.2 (also termed SL9266) is part of an essential *cis*-acting replication element (CRE) located in the NS5B coding sequence (Fig. 1A; Romero-López and Berzal-Herranz 2017).

The distal contact established between 3′X and 5BSL3.2 (Fig. 1A) has been reported to be essential for virus replication (Friebe et al. 2005; You and Rice 2008; Murayama et al. 2010) and translation (Romero-López and Berzal-Herranz 2012; Tuplin et al. 2015). This contact occurs through base-pairing of two 7-nt-long 3′X and 5BSL3.2 sequence motifs named *k* and *k*′, respectively. The *k* motif of domain 3′X is contained within a 16-nt palindromic tract termed dimer linkage sequence (DLS) (Fig. 1A,B; Supplemental Fig. S1).

The DLS promotes dimerization of viral genomes in vitro (Cristofari et al. 2004; Ivanyi-Nagy et al. 2006; Shetty et al. 2010; Cantero-Camacho and Gallego 2015; Cantero-Camacho et al. 2017) through the formation of an intermolecular kissing loop contact between two palindromic loops (Shetty et al. 2010). In addition, the efficiency of dimer formation is controlled by distant regions of the HCV genome including the CRE and the IRES (Romero-López et al. 2017). The DLS motif is absolutely conserved among the different HCV isolates (Yamada et al. 1996), and mutations in the DLS palindrome affecting dimerization have been shown to abrogate viral replication (Masante et al. 2015). Since the *k* sequence is included in the DLS, the establishment of the distal contact with 5BSL3.2 and the formation of intermolecular homodimers must be mutually exclusive. This suggests a switch-type function for the DLS/*k* motif of the HCV genome.

NMR spectroscopy and small angle X-ray scattering (SAXS) experiments have been used to study the solution structure of domain 3′X, both in isolation (Cantero-Camacho and Gallego 2015; Cantero-Camacho et al. 2017; Kranawetter et al. 2017) and complexed with 5BSL3.2 (Cantero-Camacho and Gallego 2018). When isolated, the domain adopts a conformation comprising two stem–loops, SL1′ and SL2′, stabilized by coaxial stacking (Fig. 1B; Cantero-Camacho and Gallego 2015; Cantero-Camacho et al. 2017; Kranawetter et al. 2017). This fold exposes a palindromic tetranucleotide sequence—the four central nt of the DLS—in the apical loop of subdomain SL2′, promoting dimerization of the 3′X domain at higher ionic strength and/or RNA concentration (Cantero-Camacho and Gallego 2015; Cantero-Camacho et al. 2017). In the two-stem conformation, the *k* motif is almost completely buried in the upper double helix of subdomain SL2′ and apparently unable to form a kissing loop complex with the *k*′ complementary motif within the 5BSL3.2 apical loop (Fig. 1B). An alternative three-stem domain structure where the *k* sequence is exposed in the terminal loop of hairpin SL2 was proposed on the basis of chemical modification experiments (Blight and Rice 1997; Ito and Lai 1997; Dutkiewicz and Ciesiolka 2005; Ivanyi-Nagy et al. 2006; Romero-López et al. 2014). This led to the suggestion that the two-stem and three-stem folds of domain 3′X were part of an RNA-based switch signaling the transition between the replication, translation, and possibly genome packaging processes of the virus (Dutkiewicz and Ciesiolka 2005; Shetty et al. 2010, 2013; Tuplin et al. 2012; Palau et al. 2013; Romero-López et al. 2014). However, NMR spectroscopy experiments indicated that the 3′X domain retained the two-stem–loop conformation upon binding to 5BSL3.2. Complex formation implied a conformational change in just the upper portion of subdomain SL2′ where the nt of the *k* motif are base-paired, involving disruption of those base pairs and formation of new pairs with the *k*′ motif nt of the 5BSL3.2 loop (Cantero-Camacho and Gallego 2018).

To determine the role of 3′X domain structure on the virus cycle and further clarify the mechanism of the proposed conformational switch, we have carried out a structural and functional analysis of this domain including several mutations that either stabilized the two-stem conformation observed for the wild-type domain, or favored the alternative three-stem structure (Fig. 1B). The effect of these mutations on the conformation and stability of the domain as well as on its capacity to dimerize and bind 5BSL3.2 was assessed with NMR spectroscopy, gel electrophoresis, and thermal denaturation experiments. In parallel, the impact of the mutations on the translation and replication activities of the virus was evaluated in cell culture assays. The results indicate that the conformation and the homo- and hetero-association capacities of the 3′X domain are directly related and have a marked influence on the translation and replication processes of the virus.

## RESULTS

### Design of mutants modifying the structure of the 3′X domain

To study the effect of 3′X domain structure on viral function, we designed six mutants that did not affect the universally conserved DLS/*k* tract and were predicted by RNA-folding algorithms to modify the conformation of the domain. Three of them, U_3_C, U_55_C, and the double mutant U_3_C/U_55_C, stabilized the two-stem structure observed by NMR and SAXS experiments (Cantero-Camacho and Gallego 2015; Cantero-Camacho et al. 2017) by converting the U3:G53 and/or G1:U55 pairs at the base of subdomain SL2′ into G:C pairs (Fig. 1B). In contrast, mutants U_3_G, G_50_C/C_52_G, and U_3_G/G_50_C/C_52_G were designed to destabilize the two-stem conformation by disrupting the U3:G53, G4:C52, and C6:G50 pairs of subdomain SL2′. These latter mutants were predicted by RNA folding algorithms to form the three-stem structure formerly proposed on the basis of chemical modification techniques (Fig. 1B; Blight and Rice 1997; Ito and Lai 1997; Dutkiewicz and Ciesiolka 2005; Ivanyi-Nagy et al. 2006; Romero-López et al. 2014).

In addition, the previously studied C_29_G/A_31_U mutant (Cantero-Camacho and Gallego 2018) was included in this work. In this mutant, the C_29_UAG_32_ sequence of the SL2′ apical tetraloop was changed to G_29_UUG_32_, so that the DLS palindrome was disrupted without affecting the *k* motif involved in 5BSL3.2 association (Fig. 1B). NMR spectroscopy and gel electrophoresis data indicated that this mutant adopted the two-stem conformation of the wild-type sequence and exhibited the same capacity to bind 5BSL3.2, but was unable to homodimerize (Cantero-Camacho and Gallego 2018). The effect of this mutation on the translation and replication capacities of the virus was also analyzed and compared with the effects brought about by the six structural mutants.

### Solution structure of 3′X domain mutants in the monomeric state

**FIGURE 2.**
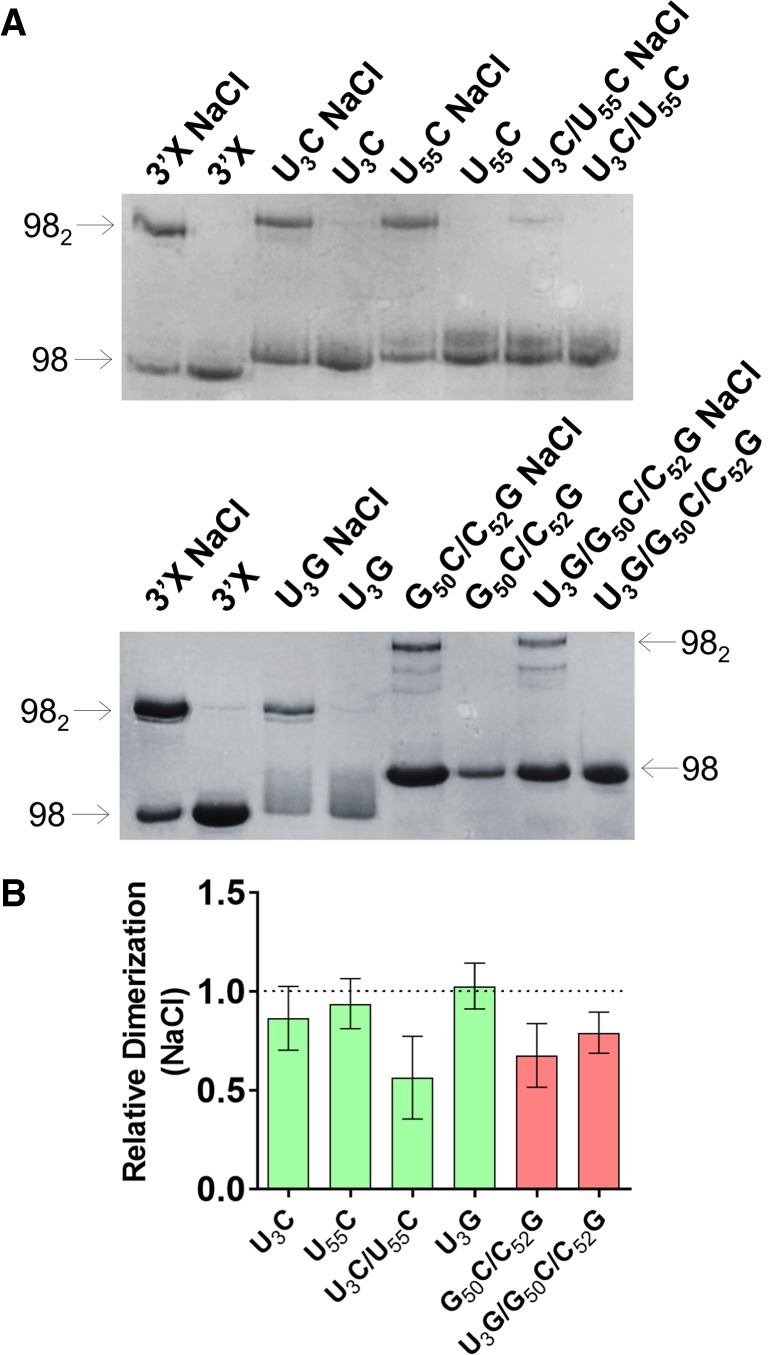
Gel electrophoresis analysis of 3′X domain molecules as a function of ionic strength. (*A*) Representative native gels comparing the electrophoretic mobility of wild-type and U_3_C, U_55_C, U_3_C/U_55_C, U_3_G, G_50_C/C_52_G, and U_3_G/G_50_C/C_52_G mutant domain molecules, previously folded in the absence or presence of 100 mM NaCl. The arrows indicate the position of the monomer (98) and homodimer (98_2_) species. (*B*) Quantification of the homodimerization yield at 100 mM NaCl of mutant molecules relative to the wild-type domain, which was assigned a value of 1. The bars represent the average and standard deviation of three independent experiments. Mutants experimentally verified to adopt the wild-type two-stem conformation are represented in green, whereas mutants adopting a different structure are indicated in red. Conditions: 10–40 µM RNA, TB running buffer.

The solution structure of the mutant 3′X domain monomers was first assayed by native gel electrophoresis (Fig. 2; Supplemental Fig. S2). The results indicated that the monomeric species of the U_3_C, U_55_C, and U_3_C/U_55_C domain mutants stabilizing the two-stem conformation had an electrophoretic mobility comparable to that of the wild-type sequence. Mutant U_3_G migrated similarly to the sequences favoring the two-stem conformation, although forming a smeared band. In contrast, the monomeric species of the double (G_50_C/C_52_G) and triple (U_3_G/G_50_C/C_52_G) mutants destabilizing the two-stem conformation migrated more slowly (Fig. 2; Supplemental Fig. S2). This indicated that the monomeric conformation of the U_3_C, U_55_C, U_3_C/U_55_C, and U_3_G mutants was similar to that of the wild-type sequence, whereas the destabilizing double and triple mutants likely adopted a different solution structure.

**FIGURE 3.**
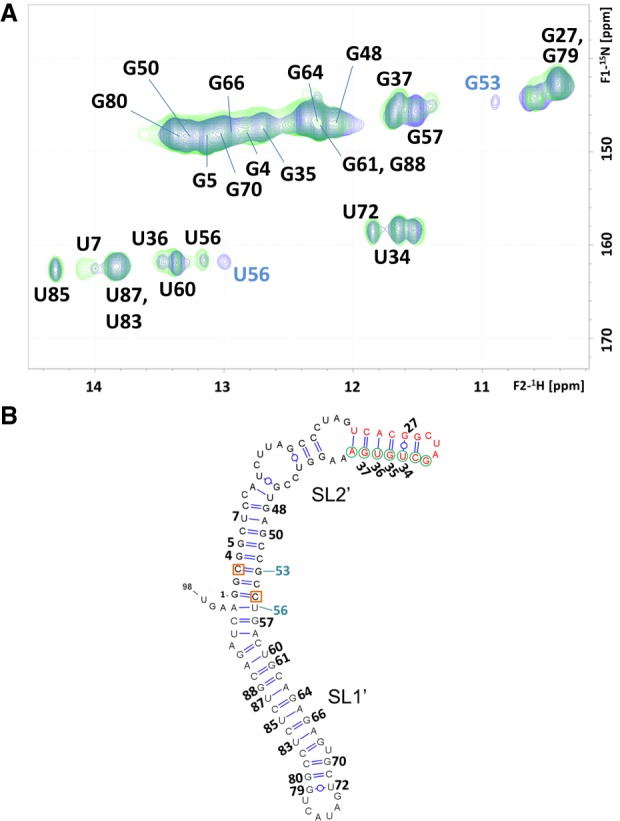
NMR spectroscopy analysis of the U_3_C/U_55_C 3′X mutant. (*A*) ^1^H–^15^N HSQC spectrum of U_3_C/U_55_C at low ionic strength (green), superposed with that of the wild-type 3′X domain (blue) acquired under the same temperature and ionic conditions. All assignments appear in black except for those of wild-type G53 and U56 HN crosspeaks, which do not overlap with U_3_C/U_55_C mutant signals and are labeled in blue. In the wild-type domain, the U56 signal is doubled due to conformational exchange. (*B*) Secondary structure model of the U_3_C/U_55_C mutant domain supported by the NMR data. DLS nt are depicted in red, *k* motif nt are indicated with green circles, and the U_3_C and U_55_C mutations stabilizing the lower stem of subdomain SL2′ are marked with red squares. Residues with assigned HN resonances are numbered. Conditions: 64 µM U_3_C/U_55_C, 0 mM NaCl/MgCl_2_, 27°C.

These observations were corroborated by an NMR analysis of the mutant conformations under conditions of low ionic strength and low RNA concentration, previously shown to favor the presence of monomers (see Fig. 2; Supplemental Fig. S2; Cantero-Camacho and Gallego 2015; Cantero-Camacho et al. 2017). In a ^1^H–^15^N HSQC NMR spectrum, each HN imino crosspeak indicates the presence of a base pair, and the crosspeak frequencies depend on the nature of the bases and their neighboring nt. We found a close match between the imino crosspeaks of the U_3_C/U_55_C mutant and those of the wild-type sequence, with the exception of the wild-type G53 and U56 imino crosspeaks (Fig. 3A). U3:G53 was replaced by C3:G53 in the mutant domain, whereas the U56 imino was perturbed because U56:A95 neighbors the terminal G1:U55 pair, which was mutated to G1:C55 (Fig. 1B). These imino assignments were based on analyses of ^1^H–^1^H NOESY and TOCSY spectra supported by previous wild-type domain and subdomain molecule analyses (Cantero-Camacho and Gallego 2015). Except for the above changes, the NOE crosspeak patterns of U_3_C/U_55_C (Supplemental Fig. S3) were also found to be analogous to those of the wild-type domain and subdomain constructs. This indicated that the stabilizing U_3_C/U_55_C mutant formed a two-stem monomer conformation similar to that adopted by the wild-type sequence, as depicted in Figure 3B.

We also carried out a detailed NMR analysis of U_3_G, since this mutant was designed to promote the three-stem conformation, but had an electrophoretic behavior different to the U_3_G/G_50_C/C_52_G and G_50_C/C_52_G sequences (Fig. 2; Supplemental Fig. S2). In this mutant, a new U55:A96 Watson–Crick pair was detected, connected with NOEs to U56:A95. Likewise, a new U:G HN3 imino peak appeared, tentatively assigned as U98 (Supplemental Figs. S4, S5). Since U55:A96 and U56:A95 are the only possible consecutive A:U pairs in the entire domain, these observations clearly indicate an extension of the SL1′ stem, induced by the disruptive U3G mutation at the base of the SL2′ helix (see Supplemental Fig. S4B). Despite these changes, the chemical shifts and NOE patterns of most subdomain SL2′ residues were similar to those detected in the wild-type sequence (Supplemental Figs. S4, S5). Therefore, the NMR results indicated that mutant U_3_G adopted a two-stem conformation comprising an extended SL1′ subdomain, as illustrated in Supplemental Figure S4B.

The imino resonances and NOE crosspeaks detected in the spectra of U_3_G/G_50_C/C_52_G (Supplemental Figs. S6, S7) indicated that this mutant formed, like U_3_G, an extended SL1′ subdomain, which is present in the three-stem conformation predicted for this triple mutant sequence (Fig. 1B). However, the resonances and NOEs typical of the SL2′ subdomain, observed in the wild-type sequence and mutants U_3_C/U_55_C and U_3_G, were not detected in this mutant. Furthermore, several new signals appeared in the HSQC and NOESY spectra (Supplemental Figs. S6, S7). These results indicated that U_3_G/G_50_C/C_52_G adopted a conformation, or mixture of conformations, different to that formed by the wild-type sequence or the U_3_C/U_55_C and U_3_G mutants. The appearance of multiple resonances prevented us from extracting further conclusions regarding the solution structure of this mutant.

On the other hand, analyses of the homonuclear spectra of the remaining mutants, U_3_C, U_55_C, and G_50_C/C_52_G, confirmed that the first two adopted a two-stem conformation similar to that of the wild-type and mutant U_3_C/U_55_C domains, whereas G_50_C/C_52_G, like the triple U_3_G/G_50_C/C_52_G mutant, adopted a different conformation (data not shown).

### Thermal stability of wild-type and mutant 3′X monomers

**FIGURE 4.**
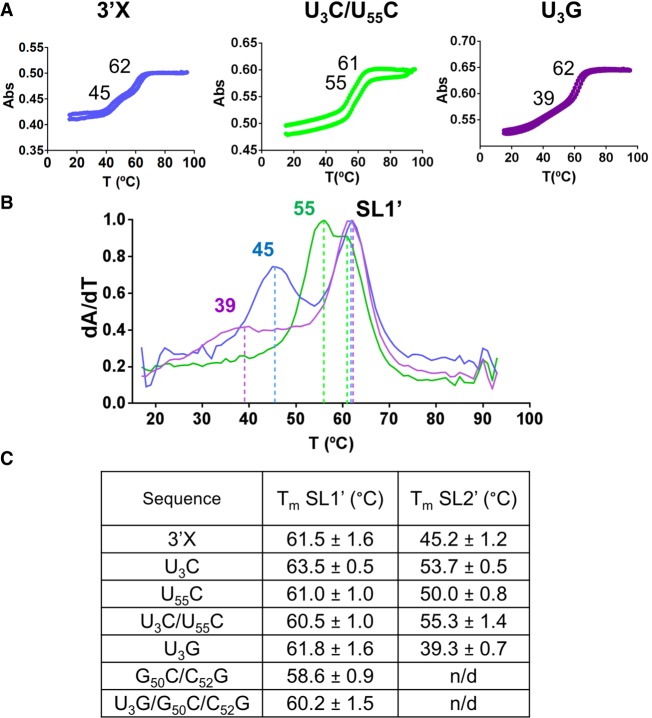
Thermal stability of subdomains SL1′ and SL2′ in wild-type and mutant 3′X domain molecules. (*A*) Representative UV-monitored thermal denaturation and renaturation curves of wild-type and mutant U_3_C/U_55_C and U_3_G domain molecules. The average melting temperatures of subdomains SL1′ and SL2′ are indicated on each graph. (*B*) Superposition of the first derivative of absorbance as a function of temperature for wild-type, U_3_C/U_55_C and U_3_G domain molecules. The color code is the same as that used in *A*. (*C*) Average melting temperatures of subdomains SL1′ and SL2′ of wild-type and mutant 3′X domain molecules. Conditions: 10 mM sodium phosphate (pH 6.0), 0.1 mM EDTA, and 0 mM NaCl/MgCl_2_.

We also assessed the stability of the wild-type and mutant domains with UV thermal denaturation experiments, carried out in conditions of low RNA concentration and low ionic strength favoring the presence of monomeric species (Fig. 2; Cantero-Camacho and Gallego 2015; Cantero-Camacho et al. 2017). Most sequences exhibited reversible curves comprising two sequential transitions. The higher-temperature transition was detected at a relatively narrow range of temperatures (between 59°C and 64°C; Fig. 4; Supplemental Fig. S8). Experiments involving an isolated subdomain molecule (data not shown) indicated that this transition corresponded to the melting of subdomain SL1′, which was formed by all sequences according to the NMR data (see above). In contrast, the temperature of the first transition was markedly influenced by the different sequences. The U_3_C, U_55_C and U_3_C/U_55_C mutations stabilizing subdomain SL2′ (Fig. 1B) increased the temperature of this transition between 6°C and 10°C relative to the wild-type value (45°C) (Fig. 4; Supplemental Fig. S8). On the contrary, the temperature of the transition decreased by 6°C (to 39°C) in mutant U_3_G, which destabilized subdomain SL2′ but still formed the two-stem conformation as indicated by the NMR data (Supplemental Fig. S4). Mutants U_3_G/G_50_C/C_52_G and G_50_C/C_52_G, which according to the NMR results did not adopt the two-stem conformation, failed to exhibit a clear lower-temperature transition (Supplemental Fig. S8). This indicated that in these cases the conformation adopted by the first 50 nt of the domain was unstable, in agreement with the observation of multiple resonances in the NMR spectra of these two sequences (Supplemental Figs. S6, S7).

### Homodimerization of mutant 3′X domains

All mutants containing the wild-type DLS motif formed homodimers in conditions of higher ionic strength (Fig. 2; Supplemental Fig. S2), as detected previously for the wild-type 3′X sequence (Shetty et al. 2010; Cantero-Camacho and Gallego 2015; Cantero-Camacho et al. 2017). However, the electrophoresis experiments revealed a number of important differences. First, in the presence of either 100 mM NaCl or 2 mM MgCl_2_ the electrophoretic mobility of the dimers formed by the mutants adopting the two-stem conformation was similar to that of the wild-type dimers. In contrast, the mobility of the dimers formed by the destabilizing G_50_C/C_52_G and U_3_G/G_50_C/C_52_G mutants was strikingly reduced (Fig. 2; Supplemental Fig. S2). This indicated that the structure adopted by these homodimers was markedly different relative to wild-type, and was likely multibranched (Lilley 2008). Likewise, the homodimerization efficiency of mutants G_50_C/C_52_G and U_3_G/G_50_C/C_52_G was reduced with respect to wild-type or mutant sequences adopting the two-stem conformation (Fig. 2B; Supplemental Fig. S2B). This was not surprising, as 3′X domain dimerization involves the formation of an intermolecular kissing loop contact between two palindromic DLS tetranucleotides exposed in the apical SL2′ loop of the two-stem conformation (Fig. 1B; Cantero-Camacho and Gallego 2015; Cantero-Camacho et al. 2017), which was not detected by NMR spectroscopy in the G_50_C/C_52_G and U_3_G/G_50_C/C_52_G mutants (Supplemental Figs. S6, S7).

After the initial loop–loop contact is established, extended homodimers comprising an intermolecular SL2′ double-helix can be formed by the wild-type domain, as shown by NMR and SAXS data (Cantero-Camacho and Gallego 2015; Cantero-Camacho et al. 2017). Since 3′X–3′X kissing dimers are stabilized by Mg^2+^ (Shetty et al. 2010), extended homodimers are predominantly formed in the absence of Mg^2+^ and presence of Na^+^ (Cantero-Camacho and Gallego 2015; Cantero-Camacho et al. 2017). Under these conditions (100 mM NaCl), the patterns of crosspeaks in the HSQC spectra of mutants U_3_G and U_3_C/U_55_C underwent little changes relative to those detected in the absence of added salts (Supplemental Figs. S9A, S11A). This indicated that these mutants likely formed extended symmetric dimers similar to those described for the wild-type sequence (Cantero-Camacho and Gallego 2015), particularly since the secondary structure of these dimers is very similar to that of the monomers with the exception of the continuous 16-bp duplex formed by the self-complementary DLS nt (Supplemental Fig. S9B). Likewise, since kissing dimers are not predominant in the presence of NaCl, the differences in dimerization efficiency between two-stem and three-stem mutants were reduced relative to those observed at 2 mM MgCl_2_ (compare Fig. 2B; Supplemental Fig. S2B). In addition, the U_3_C/U_55_C double mutant tended to form homodimers less efficiently than the U_3_C, U_55_C or U_3_G mutants (Fig. 2B), likely because formation of the extended dimers implies melting of SL2′ hairpins (to form intermolecular SL2′ duplexes), and the thermal stability of the SL2′ subdomain is increased in this mutant (Fig. 4).

### 5BSL3.2 binding capacity of 3′X mutant domains

Several groups have shown that the 7-nt *k* tract in the 3′X DLS forms a distal base-pairing interaction with a complementary *k*′ sequence located in the apical loop of domain 5BSL3.2 (Friebe et al. 2005), which modulates the replication and translation processes of HCV (Friebe et al. 2005; You and Rice 2008; Murayama et al. 2010; Tuplin et al. 2012, 2015). We recently detected the formation of inter-domain *k*–*k*′ base-pairing in the 3′X-5BSL3.2 complex by NMR spectroscopy, and showed that the interaction involved a conformational change in the upper portion of subdomain SL2′, so that the 3′X domain retained the two-stem conformation upon binding to the 5BSL3.2 stem–loop (Cantero-Camacho and Gallego 2018).

**FIGURE 5.**
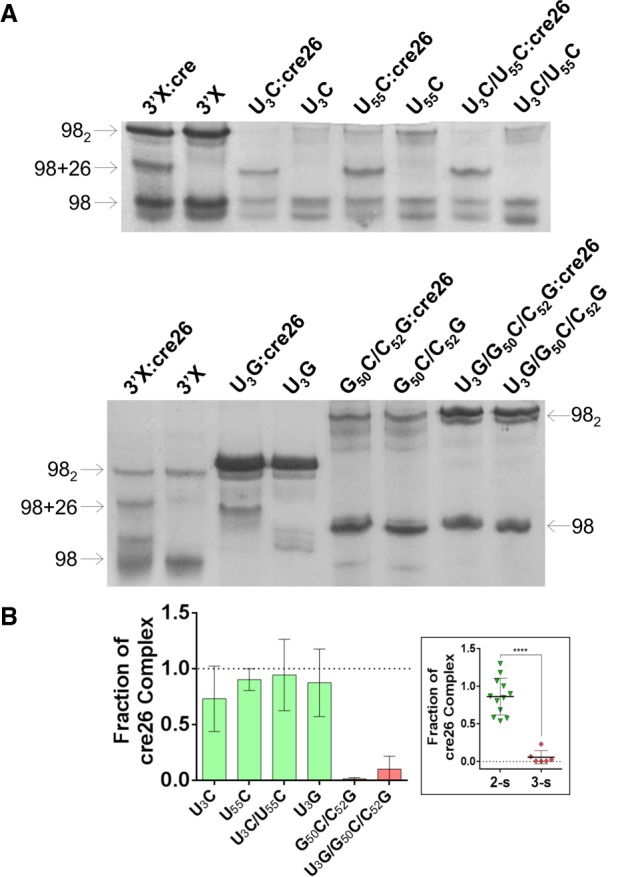
Gel electrophoresis analysis of 3′X domain molecules and their complexes with the 5BSL3.2 hairpin cre26. (*A*) Native gels comparing the electrophoretic mobility of wild-type and mutant U_3_C, U_55_C, U_3_C/U_55_C, U_3_G, G_50_C/C_52_G, and U_3_G/G_50_C/C_52_G 3′X domain molecules in the absence and presence of one molar equivalent of cre26. The arrows indicate the position of the monomer (98), homodimer (98_2_), and heterodimer (98 + 26) species. (*B*) Quantification of the interaction between mutant 3′X domain molecules and cre26, obtained by measuring the fraction of 3′X:cre26 complexes (98 + 26) relative to total 3′X RNA. The results were normalized with respect to the wild-type sequence, which was assigned a value of 1, and the bars represent the average and standard deviation of three independent experiments. Mutants experimentally verified to adopt the wild-type two-stem conformation are represented in green, whereas mutants adopting a different structure are indicated in red. In the scatter plot shown in the *inset*, the individual values of the two-stem and three-stem mutants are indicated, together with means and standard deviations. The differences between two-stem and three-stem mutants are significant in all cases (*P* < 0.005). Conditions: 10–40 µM RNA, TBM running buffer (2 mM MgCl_2_).

In view of these findings, we used native gel mobility shift assays to assess the 5BSL3.2 binding capacity of the 3′X mutants, none of which involved changes in the *k* tract of the 3′X domain (Fig. 5). The results indicated that all mutants forming the wild-type conformation, namely U_3_C, U_55_C, U_3_C/U_55_C, and U_3_G, were able to associate to domain 5BSL3.2. In contrast, mutants G_50_C/C_52_G and U_3_G/G_50_C/C_52_G, adopting a different conformation, did not bind 5BSL3.2 or bound it very weakly (Fig. 5). These results indicated that the two-stem conformation adopted by the wild-type sequence is required for 5BL3.2 binding, even though the alternative conformation would presumably expose the *k* sequence motif in the apical loop of the SL2 subdomain (Fig. 1B).

Using NMR spectroscopy, we also assessed the mechanism of 5BSL3.2 association of the U_3_C/U_55_C and U_3_G 3′X mutants. No significant chemical shift changes were observed in the HN imino crosspeaks of any of the two sequences upon the addition of unlabeled cre26, a 26-nt-long RNA molecule comprising the apical portion of the 5BSL3.2 domain (Fig. 1B; Supplemental Figs. S10A, S11B). This indicated that both sequences retained the SL1′ and SL2′ conformation upon binding to cre26, as previously shown for mutant C_29_G/A_31_U (Cantero-Camacho and Gallego 2018). However, the HN imino crosspeaks of nt G35, U36, and G37 within the *k* tract of the 3′X domain, lost their respective interactions with C26, A25, and C24 in the HNN-COSY spectra of the complexes, and at the same time, the G27 and U34 imino resonances weakened (Supplemental Figs. S10A, S11B, green labels). These changes can be attributed to the formation of an inter-domain 3′X:cre26 duplex where these U_34_GUG_37_ nt of the *k* sequence motif pair in antiparallel orientation with the complementary *k*′ motif nt of the unlabeled cre26 sequence (Supplemental Fig. S10C; Cantero-Camacho and Gallego 2018).

### The conformation of the 3′X domain modulates the activity of the IRES

**FIGURE 6.**
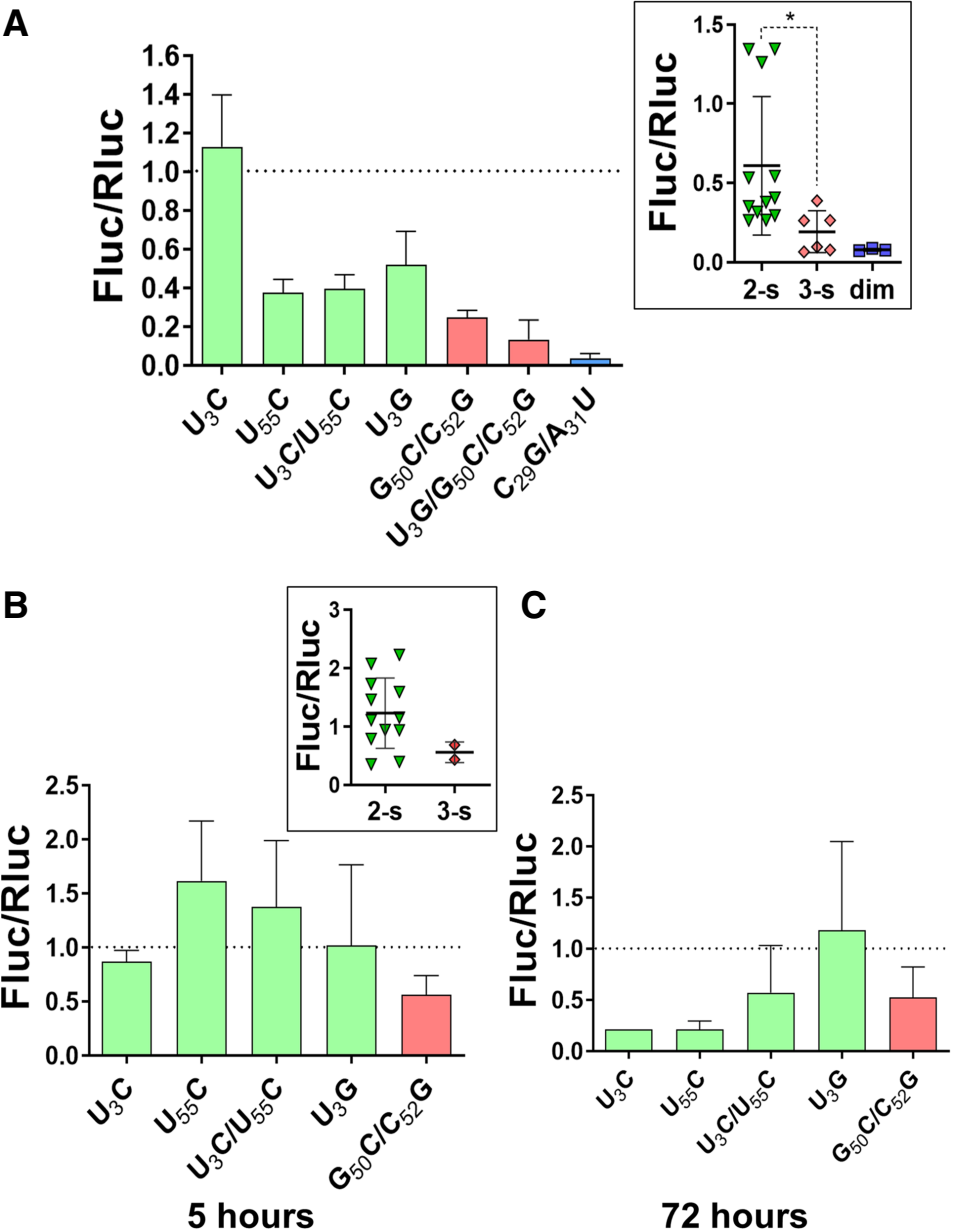
Effect of 3′X domain mutations on HCV translation and replication. (*A*) Huh-7 cells were cotransfected with a mixture of wild-type or mutant ICU-Fluc RNA and cap-Rluc-mRNA, and IRES activity was measured as relative luciferase activity (Fluc/Rluc) as previously described (Romero-López and Berzal-Herranz 2012). The value obtained for each mutant was referred to that obtained for the wild-type molecule, which was assigned a value of 1. The bars represent the average and standard deviation of three independent experiments. (*B*,*C*) Huh-7.5 cells were electroporated with a mixture of wild-type or mutant 3′X subgenomic HCV replicon and cap-Rluc-mRNA as previously described (Fernández-Sanlés et al. 2015). Relative luciferase activity values (Fluc/Rluc) were determined at 5 h (*B*) and 72 h (*C*) after transfection, representing translation and replication activities, respectively. The Fluc/Rluc values of each mutant were referred to that obtained with the wild-type molecule, which was assigned a value of 1, and the bars represent the mean and standard deviation of an average of two independent experiments. In *A*–*C*, mutants experimentally verified to adopt the wild-type two-stem conformation are represented in green, mutants adopting a different structure are indicated in red, and the dimerization-defective C_29_G/A_31_U mutant is plotted in blue. The scatter plots shown in the *insets* display the individual values of two-stem, three-stem, and dimerization mutants, together with mean and standard deviation values.

To assess the role of 3′X domain conformation in HCV translation we constructed a series of subgenomic viral RNA mutants based on the ICU construct (Romero-López and Berzal-Herranz 2012), as described in Materials and Methods. ICU RNA molecules comprised a luciferase reporter gene mRNA flanked at its 5′ side by the HCV genomic 5′-end region including the complete IRES domain, and at 3′ by the CRE and the 3′-UTR, containing either the wild-type sequence or the U_3_C, U_55_C, U_3_C/U_55_C, U_3_G, G_50_C/C_52_G, U_3_G/G_50_C/C_52_G, and C_29_G/A_31_U 3′X domain mutations. Huh-7 cells were transfected with these RNA molecules along with cap-dependent RLuc-mRNA, and HCV translation was determined as relative luciferase activity (Fluc/Rluc). All mutants except U_3_C exhibited reduced translation levels relative to the wild-type sequence in this assay. However, U_3_C, U_55_C, U_3_C/U_55_C, and U_3_G, which adopted the two-stem wild-type conformation and bound domain 5BSL3.2, showed significantly higher translation levels than the G_50_C/C_52_G or U_3_G/G_50_C/C_52_G mutants, which formed a dissimilar conformation and exhibited reduced capacity to associate to 5BSL3.2 (Fig. 6A). The C_29_G/A_31_U DLS mutant, which adopted the two-stem conformation and had 5BSL3.2 binding capacity but was unable to homodimerize, also exhibited significantly poorer translation activity in this assay (Fig. 6A).

### The conformation of the HCV 3′X domain regulates viral translation and replication

In an attempt to further evaluate the role of 3′X domain conformation within the HCV cycle, we evaluated the impact of the different 3′X mutants on translation and replication using a replication-competent viral RNA molecule. For this purpose, the mutations were introduced in a pFK-I_389_-Fluc-NS3-3′ET plasmid encoding a subgenomic HCV replicon (Lohmann et al. 1999), and the resulting DNA vectors were transcribed in vitro to generate the corresponding replicon mutants. Huh-7.5 cells were subsequently cotransfected with each individual viral replicon mutant and cap-dependent RLuc-mRNA. At 5 h after transfection Fluc/Rluc levels, indicative of the translation capacity of the replicons, were determined (Fig. 6B). The results were consistent with those obtained with the ICU construct derivatives. The replicons with U_3_C, U_55_C, U_3_C/U_55_C, or U_3_G mutant domains, which adopted the wild-type conformation and were capable of 5BSL3.2 binding, exhibited similar or greater translation activities relative to the wild-type replicon. In contrast, the replicon with the G_50_C/C_52_G mutant domain, which did not adopt the wild-type conformation and was unable to bind domain 5BSL3.2, showed reduced translation levels (Fig. 6B).

At 72 h posttransfection, the luminescence measurements reflect the amount of viral genomic RNA (Lohmann 2009), allowing a comparison of the replication efficiency of the different HCV replicons. In this case the replicons with U_3_C, U_55_C, or U_3_C/U_55_C mutations, which stabilized the SL2′ subdomain present in the wild-type 3′X conformation, exhibited reduced replication efficiency relative to that of the wild-type sequence (Fig. 6B). The G_50_C/C_52_G double mutant, which did not adopt the wild-type 3′X conformation, also led to decreased replication activity. The replicon with the U_3_G mutant domain, which adopted the wild-type conformation but did not stabilize SL2′, was the only one that maintained wild-type replication levels (Fig. 6B).

Cell death was unexpectedly observed upon transfection of Huh-7.5 cells with the HCV replicons containing the U_3_G/G_50_C/C_52_G triple mutation or the C_29_G/A_31_U DLS mutation. This effect impeded a reliable assessment of the impact of these two mutations on HCV replicon translation and replication.

## DISCUSSION

**FIGURE 7.**
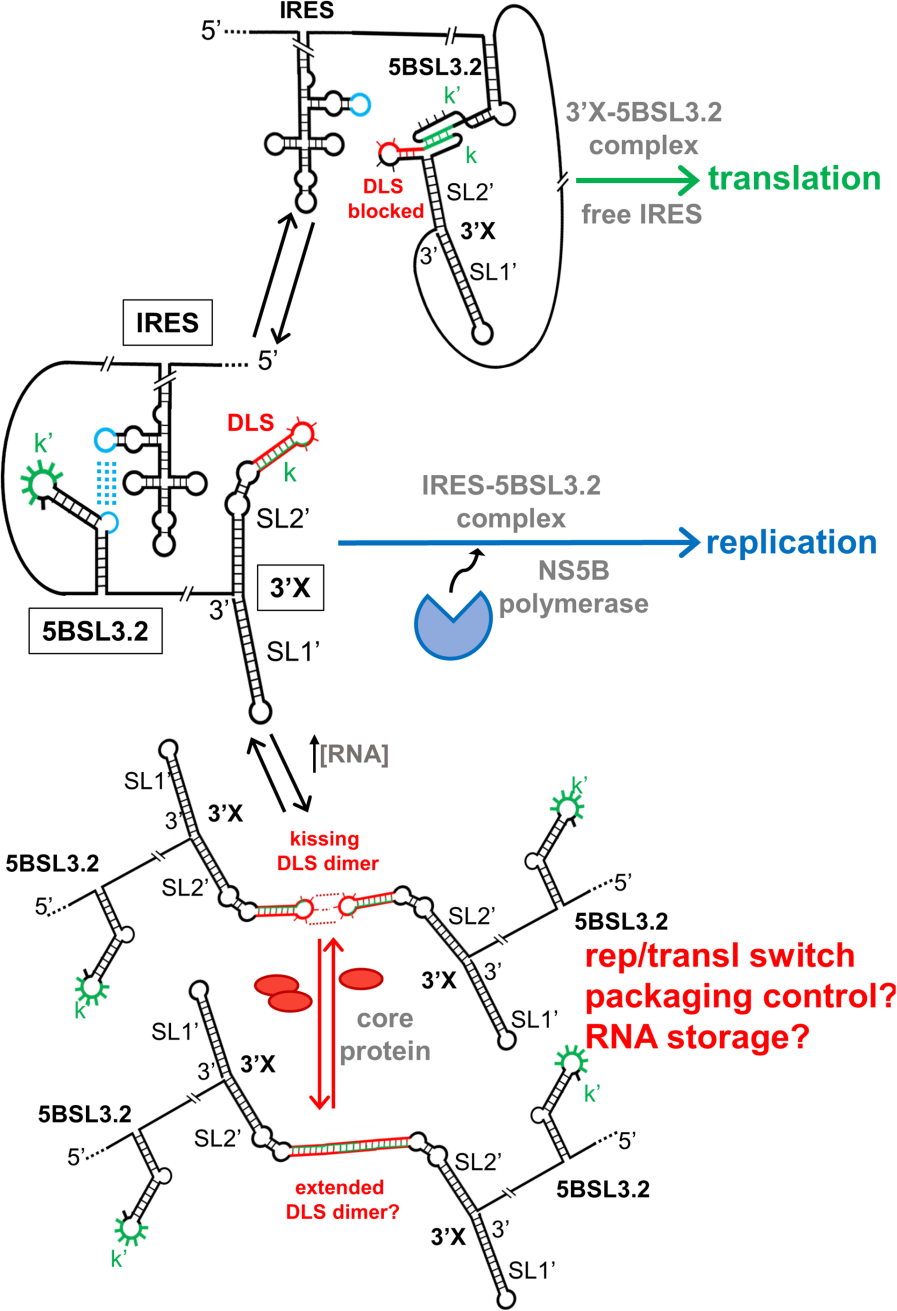
Scheme summarizing the structure and functional interactions of the 3′X domain of HCV, together with their possible impact on the virus life cycle. When the distal contact between the 3′X and 5BSL3.2 domains is established, the IRES is free and can initiate translation. In the absence of this interaction, the 5BSL3.2 domain is complexed with the IRES and synthesis of the negative-sense strand starts from the 3′X domain. At higher viral RNA concentrations, 3′X domain homodimers are formed through kissing loop contacts between palindromic DLS loops. These homodimers are involved in regulating the switch between translation and replication. They may also act as intermediary species in the process of packaging, or alternatively as a means to store virus RNA in the infected cell. For simplicity, the SL9005 domain is not shown.

By combining structural and functional data from different mutants, we show that the conformation adopted by the 3′X domain of HCV comprises two SL1′ and SL2′ stems, and propose the molecular mechanisms by which this structure modulates the replication and translation processes of the virus (Fig. 7). In addition, our results explain the high degree of sequence conservation detected for this domain (Yamada et al. 1996), indicative of an essential regulatory role in the virus replication cycle.

Only the two-stem conformation of the 3′X domain allowed the distal interaction with domain 5BSL3.2 to be established (Fig. 5). This contact has been reported to be required either for virus replication (Friebe et al. 2005; You and Rice 2008; Murayama et al. 2010) or translation (Romero-López and Berzal-Herranz 2012; Tuplin et al. 2015). Our results show that in both the ICU and replicon systems, the mutants that adopted the wild-type conformation and were capable of binding 5BSL3.2 exhibited improved translation activities relative to mutants that formed a different structure and were unable to recognize 5BSL3.2 (Fig. 6A,B). These results are consistent with previous reports indicating a role of the 3′X-5BSL3.2 contact in translation. These reports showed that the inhibitory effect of CRE (containing the 5BSL3.2 domain) on translation diminished in the presence of the 3′-UTR (Romero-López and Berzal-Herranz 2012), and that blockage of the 3′X-5BSL3.2 contact specifically inhibited the translation process (Romero-López and Berzal-Herranz 2012; Tuplin et al. 2015). According to our model, formation of the 3′X-5BSL3.2 complex likely obstructs the distal interaction between 5BSL3.2 and the viral IRES (Romero-López and Berzal-Herranz 2017), allowing the latter domain to initiate translation (Fig. 7).

The fact that only the mutants adopting the two-stem conformation were able to bind 5BSL3.2 is apparently surprising, since the alternative conformation predicted to be favored by the SL2′-destabilizing mutants exposes the *k* sequence motif involved in the base-pairing interaction with the *k*′ motif of 5BSL3.2 (Fig. 1B). However, we previously showed by NMR spectroscopy that 3′X-5BSL3.2 complex formation did not imply a transition from the two-stem conformation, but a reorganization of the upper portion of subdomain SL2′ to allow formation of the inter-domain *k*–*k*′ duplex (Fig. 7; Supplemental Fig. S10C; Cantero-Camacho and Gallego 2018). This association mechanism has been confirmed here with the U_3_C/U_55_C and U_3_G mutants (Supplemental Figs. S10, S11B).

Another important feature of the two-stem functional conformation of the 3′X domain is the exposure of a palindromic sequence (DLS) in the apical portion of subdomain SL2′ (Fig. 1B; Cantero-Camacho and Gallego 2015; Cantero-Camacho et al. 2017). This allowed the wild-type sequence and the mutants adopting this conformation to readily form homodimers at higher ionic strength or in the presence of Mg^2+^ ions (Fig. 2; Supplemental Fig. S2). The exact mechanism by which 3′X dimerization influences the viral cycle is not yet clear, but the 16-nt DLS tract driving dimerization is absolutely conserved in all viral isolates (Yamada et al. 1996), pointing to a key functional role. In this respect, the dimerization-defective C_29_G/A_31_U mutant had very poor translation activity (Fig. 6A), and the mutants that destabilized the wild-type structure and showed low homodimerization capacity also exhibited lower translation and replication efficiencies (Fig. 6). These results are in good agreement with previous reports indicating that viral RNA dimerization controls the replication and translation processes of the virus (Ivanyi-Nagy et al. 2006; Shetty et al. 2010; Masante et al. 2015). Likewise, we have recently reported that genomic HCV dimerization is controlled by the CRE and the IRES domains, and have identified specific nt within essential structural elements of the IRES and 5BSL3.2 that are responsible for dimerization regulation (Romero-López et al. 2017). This suggests that a dynamic network of RNA–RNA interactions controls essential viral processes (Romero-López and Berzal-Herranz 2017).

It is also possible that the DLS plays a role related to packaging. The 3′-UTR of HCV acts as a *cis*-acting element for core binding and packaging (Shi et al. 2016), along with other conserved domains within the RNA genome (Stewart et al. 2016; Li et al. 2018). The packaging signals of retroviruses like HIV-1, which pack two copies of viral RNA genome, also comprise dimerization sequences (Moore and Hu 2009). HCV virions are widely thought to contain one copy of genomic RNA, but the NS5B polymerase generates subgenomic molecules (Shimizu et al. 2006; Shi and Suzuki 2018). In this context, the presence of the DLS at the 3′ end of the viral genome might have evolved as a mechanism to ensure that only full-length RNA molecules are packaged into virions (Cantero-Camacho et al. 2017). Experiments involving full-length viruses will be necessary to test this possibility.

A switching function has been attributed to the 3′X domain, which would allow the individual genomic RNA molecules to alternate between the presumed mutually exclusive processes of replication, translation, and packaging (Dutkiewicz and Ciesiolka 2005; Shetty et al. 2010, 2013; Tuplin et al. 2012; Palau et al. 2013; Romero-López et al. 2014; Holmstrom et al. 2017). Based on our results, this switching mechanism is not based on a transition between two different domain conformations, but is likely due to the fact that the DLS palindrome is blocked when the DLS–5BSL3.2 contact is established and vice-versa, the DLS–5BSL3.2 interaction does not likely occur in the context of a DLS–DLS dimer (see Fig. 5). This points to 5BSL3.2 as another important player in this regulatory process (Romero-López and Berzal-Herranz 2017). According to this model (Fig. 7), local genome RNA concentration would be the key factor triggering the transition between DLS–5BSL3.2 complexes and DLS–DLS homodimers, as we had previously hypothesized (Romero-López and Berzal-Herranz 2017). The DLS–5BSL3.2 contact, which is compatible with translation as discussed above, is likely the preferred conformation of genomic RNA monomers. 3′X–3′X homodimers, on the other hand, have been reported to be optimal templates for the viral NS5B polymerase (Masante et al. 2015). This model has some analogies with the switching mechanism proposed for the dimerization sequence of HIV-1, which is also sequestered through base-pairing with a distal sequence in the monomeric conformation favoring translation (Keane and Summers 2016). In addition, it has been reported that the viral core protein has less affinity for 3′-UTR sequences in the presence of 5BSL3.2 (Shi and Suzuki 2018), so that in the case of HCV, DLS–DLS dimers may act as intermediary species in the process of packaging.

The thermal stability of subdomain SL2′ may be important for the replication process. The mutants that stabilized SL2′ exhibited higher translation levels in the replicon system but reduced replication activities. In contrast, the U_3_G mutant, which decreased the thermal stability of subdomain SL2′ while maintaining the two-stem conformation, exhibited wild-type translation and replication values (Fig. 6B,C). The influence of SL2′ on replication is not surprising, as in the vicinity of this subdomain the terminal unpaired nt at the 3′ end of the SL1′ stem favor de novo initiation of RNA synthesis by the viral NS5B polymerase (Lohmann 2013).

Altogether, our results indicate that sequence is likely important to preserve the proper stability of the conformation and to allow a precise equilibrium between translation and replication. Nucleotide changes disturbing the DLS sequence, the two-stem structure or the thermal stability of the domain resulted in less homodimerization or 5BSL3.2 association capacity, and poorer replication and translation levels. This provides a rationale for the stringent conservation detected for the 3′X domain, and particularly for the 55 nt forming subdomain SL2′ (Yamada et al. 1996).

In conclusion, the functional conformation of the terminal 3′X domain of the HCV genome comprises two stems. This structure allows the establishment of a distal contact with domain 5BSL3.2 in the ORF, required for translation, or alternatively the formation of kissing domain homodimers, which are likely involved in regulating the switch between translation and replication.

## MATERIALS AND METHODS

### Sequences

The sequences analyzed in this study correspond to HCV genotype 1b, isolate Con1 (GenBank AJ238799), and were obtained from vector pFK-I389-Fluc-NS3-3′ET (Lohmann et al. 1999).

### Secondary structure predictions

Secondary structure models were generated with the RNAfold (http://rna.tbi.univie.ac.at/) (Lorenz et al. 2011) and/or mfold (http://mfold.rna.albany.edu) (Zuker 2003) web servers. The structures were drawn using VARNA (http://varna.lri.fr/) (Darty et al. 2009).

### Preparation of RNA samples for NMR spectroscopy and gel electrophoresis experiments

The 98-nt-long U_3_C, U_55_C, U_3_C/U_55_C, U_3_G, G_50_C/C_52_G, and U_3_G/G_50_C/C_52_G mutant 3′X domain molecules (Fig. 1B; Supplemental Fig. S1) were produced by in vitro transcription with T7-RNA polymerase from ScaI-linearized pUC19 derived plasmids. These vectors were obtained by PCR site-directed mutagenesis from a pUC19 vector containing the wild-type domain (Cantero-Camacho and Gallego 2015) and verified by sequencing. The wild-type and the C_29_G/A_31_U mutant (previously identified as 3′Xm) 3′X domain molecules and the 26-nt-long cre26 molecule comprising the upper portion of the 5BSL3.2 domain (Fig. 1B; Supplemental Fig. S1) were obtained as described previously (Cantero-Camacho and Gallego 2015, 2018). For the wild-type and U_3_C/U_55_C, U_3_G, and U_3_G/G_50_C/C_52_G mutant sequences, we also generated uniformly ^13^C/^15^N-labeled transcripts using NTPs obtained from CortecNet. All constructs were purified on denaturing gels containing 20% polyacrylamide, 8 M urea. After electroelution from the gel, the RNAs were ethanol-precipitated twice and desalted with Sephadex G-25 cartridges. Prior to NMR or gel electrophoresis experiments, all samples were transferred by diafiltration into aqueous solutions containing 10 mM sodium phosphate (pH 6.0), 0.1 mM EDTA with no added salts, or additionally containing either 100 mM NaCl or 2 mM MgCl_2_. Before each NMR and gel electrophoresis experiment, the RNA samples were heated at 95°C for 5 min and immediately placed on ice for 30 min, except when analyzing extended homodimer formation at 100 mM NaCl, where they were cooled down slowly. For experiments analyzing 3′X-5BSL3.2 complex formation, the samples were snap-cooled in the absence of MgCl_2_ and then incubated with 2 mM MgCl_2_ for 150 minutes at 25°C, in the absence or presence of the interaction partner.

### NMR spectroscopy

The RNA concentration of the samples used for NMR analyses ranged between 50 and 110 µM. NMR spectra were acquired on a cryoprobe-equipped, 600 MHz Bruker Avance III spectrometer, and analyzed using TopSpin 3.5 (Bruker Biospin) and Sparky 3.110 (T.D. Goddard and D.G. Kneller, SPARKY 3, University of California, San Francisco). All U_3_C, U_55_C, U_3_C/U_55_C, U_3_G, G_50_C/C_52_G, and U_3_G/G_50_C/C_52_G mutant 3′X domain molecules were studied using unlabeled samples and two-dimensional watergate-NOESY (with 150 msec mixing time) and watergate-TOCSY experiments (60 msec mixing time) recorded in 90% H_2_O/10% D_2_O, typically at two temperatures (16°C and 27°C). The recycle delays were 1.6 and 2 sec for all homonuclear TOCSY and NOESY experiments, respectively.

The wild-type as well as U_3_C/U_55_C, U_3_G, and U_3_G/G_50_C/C_52_G mutant 3′X domain molecules were also analyzed using ^13^C/^15^N-labeled samples. For these molecules, two-dimensional ^1^H–^15^N HSQC and HNN-COSY (Dingley and Grzesiek 1998) experiments were recorded in 90% H_2_O/10% D_2_O at 27°C. For ^1^H–^15^N HSQC experiments, we acquired 128 indirect experiments with 256 scans per experiment. For HNN-COSY experiments, the delay for evolution of the ^2^J_NN_ coupling was set to 15 msec, and we collected 128 complex points in the t_1_ dimension with 360 scans for each t_1_ increment. The recycle delays were between 1.0 and 1.3 sec for all experiments with labeled samples.

#### Assignments and secondary structure determination

We have already described the assignment and secondary structure determination of the wild-type and dimerization-defective C_29_G/A_31_U 3′X domain sequences, as well as that of subdomains SL1, SL2′, and C_29_G/A_31_U-mutant SL2′ (Cantero-Camacho and Gallego 2015, 2018). The assignment of the remaining mutant sequences was based on standard analyses (Varani et al. 1996) of two-dimensional NOESY, TOCSY, HSQC, and HNN-COSY data. The assignment process was supported by comparisons with wild-type and C_29_G/A_31_U mutant 3′X domain as well as SL1, SL2′, and C_29_G/A_31_U mutant SL2′ subdomain HSQC, HNN-COSY, NOESY, and TOCSY spectra, all acquired under identical conditions. We also studied the secondary structure of the U_3_C/U_55_C, U_3_G, and U_3_G/G_50_C/C_52_G mutant domain homodimers by comparing the HSQC spectra of each sequence in the absence and presence of 100 mM NaCl, as described for the wild-type sequence (Cantero-Camacho and Gallego 2015).

#### Analysis of the distal interaction with 5BSL3.2

These studies were performed for the U_3_C/U_55_C and U_3_G domain mutants, and were based on comparing ^1^H–^15^N HSQC and HNN-COSY spectra of ^13^C/^15^N-labeled U_3_C/U_55_C and U_3_G in the absence and presence of unlabeled cre26 molecule, as previously described (Cantero-Camacho and Gallego 2018). By labeling with ^15^N/^13^C isotopes the 3′X partners, all of the HN and HNN crosspeaks corresponded to these molecules and not to unlabeled cre26. Likewise, the HN resonances detected in the mixture without a coupled N must correspond to intermolecular N–H··N hydrogen bonds.

### Gel electrophoresis experiments

Native gels were run at 4°C for 12 h under constant voltage (80 V). We used 20% 19:1 acrylamide:bisacrylamide gels and either 89 mM tris-borate (TB), or 89 mM tris-borate and 2 mM MgCl_2_ (TBM) as running buffers. These experiments involved 10–40 µM RNA samples, prepared as specified above. All gels were stained with methylene blue and destained in water. These experiments were used to measure the homodimerization and 5BSL3.2 (cre26) binding capacities of 3′X domain molecules. For each mutant, homodimerization was quantified by obtaining the fraction of 3′X molecules forming homodimers relative to total 3′X RNA present in the gel lane. Cre26 complex (heterodimer) formation was similarly obtained from the fraction of 3′X molecules forming heterodimers relative to total 3′X RNA. In both cases, the results were normalized relative to the wild-type values. Gel bands were quantified with ImageJ (Schneider et al. 2012).

### UV thermal denaturation

The thermal stability of the wild-type and mutant RNA molecules was monitored by measuring the UV absorbance at 260 nm as a function of temperature in a Varian Cary 100 UV/VIS spectrophotometer. The temperature was raised from 15°C to 95°C at a gradient of 1°C min^−1^ and subsequently decreased at the same rate to evaluate the reversibility of the process. No significant variations were detected for the *T*_m_ values when applying a temperature gradient of 0.5°C min^−1^. The experiments were carried out using 0.3–0.8 ODU ml^−1^ RNA (0.3–0.8 µM) samples dissolved in a low-ionic strength aqueous solution (10 mM sodium phosphate pH 6.0, 0.1 mM EDTA). The melting experiments were repeated three times for each molecule, and before each experiment, RNA samples were heated at 95°C for ∼5 min and immediately placed on ice for 5 min.

### DNA templates and RNA transcripts for translation and replication assays in cell culture

The translation efficiency of the 3′X domain mutants was evaluated in the context of the ICU RNA construct, which contains a luciferase reporter mRNA flanked by the HCV IRES and the 3′-end region of the viral genome (Romero-López and Berzal-Herranz 2012). The U_3_C, U_55_C, U_3_C/U_55_C, U_3_G, G_50_C/C_52_G and U_3_G/G_50_C/C_52_G and C_29_G/A_31_U 3′X domain mutations were introduced in the pGL-ICU plasmid (Romero-López and Berzal-Herranz 2012) by PCR site-directed mutagenesis following conventional procedures together with the primers listed in Supplemental Table S1, and confirmed by Sanger sequencing with specific primers (Supplemental Table S1). PCR reactions were performed with Q5 high fidelity polymerase (New England Biolabs) and 125 ng of each primer. ICU RNAs were obtained as described previously (Romero-López and Berzal-Herranz 2012).

For the evaluation of the replication and translation activity of 3′X mutants, we used a pFK-I389-Fluc-NS3-3′ET-derived plasmid encoding a bicistronic subgenomic HCV replicon composed of the HCV IRES (nt 1 to 389), a luciferase reporter gene, the IRES of the encephalomyocarditis virus directing translation of HCV nonstructural proteins NS3 to NS5B, and the 3′-UTR (Lohmann et al. 1999). To facilitate mutagenesis, a 2423-bp fragment of pFK-I389-Fluc-NS3-3′ET containing the region of interest was digested using unique Xho I and Spe I sites and subcloned into a pBluescript vector (Invitrogen). The U_3_C, U_55_C, U_3_C/U_55_C, U_3_G, G_50_C/C_52_G and U_3_G/G_50_C/C_52_G and C_29_G/A_31_U 3′X domain mutations were introduced in this vector as specified above. Plasmids encoding mutant replicons were obtained by direct subcloning of the XhoI/SpeI fragments from the pBluescript intermediates. All mutant constructs were confirmed by Sanger sequencing with specific primers (Supplemental Table S1). Subgenomic HCV replicons were obtained by in vitro transcription as previously described (Fernández-Sanlés et al. 2015). Cap-dependent Rluc-mRNA was obtained from a pRLSV40 plasmid as previously described (Romero-López et al. 2007).

### Cell lines and culture conditions

Human hepatoma Huh-7 and Huh-7.5 cell line monolayers were maintained in Dulbecco's modified Eagle medium (DMEM) supplemented with 10% heat-inactivated foetal bovine serum (Invitrogen) and 1 mM sodium pyruvate (Sigma), at 37°C in a 5% CO_2_ atmosphere.

### HCV translation and replication assays

HCV IRES-dependent translation efficiencies were determined as previously described (Romero-López and Berzal-Herranz 2012). Briefly, 48 h before Huh-7 transfection, 90,000 cells were seeded onto a 24-well plate to reach 90% confluency. A mixture containing 1 µg of wild-type or 3′X mutant ICU RNA construct containing firefly luciferase mRNA flanked by the HCV genomic ends, and 0.25 µg of cap-dependent RLuc-mRNA, was used for cell transfection with TransFectin lipid reagent (Bio-Rad). Translational efficiency was determined by measuring firefly and *Renilla* luciferase activities using the Dual-Luciferase Reporter Assay System (Promega).

The effect of 3′X domain conformation in HCV replication was assayed as previously described (Fernández-Sanlés et al. 2015). Briefly, Huh-7.5 cells were electroporated with a mixture of 5 µg of wild-type or mutant subgenomic I389-Fluc-NS3-3′ET replicon, and 200 ng of cap-dependent RLuc-mRNA. Cells were seeded onto six-well plates with 4 ml of DEMEM and 1.25% DMSO and harvested at 5 and 72 h after transfection. Firefly and *Renilla* luciferase activities were measured as described above.

## SUPPLEMENTAL MATERIAL

Supplemental material is available for this article.
